# Powassan virus infection: case series and literature review from a single institution

**DOI:** 10.1186/1756-0500-5-594

**Published:** 2012-10-30

**Authors:** Mihir Raval, Mayank Singhal, Dubert Guerrero, Augusto Alonto

**Affiliations:** 1 Sanford Hospital, Fargo, ND, USA; 2 Internal Medicine, Department of Internal Medicine, University of North Dakota, Sanford Health System, 801, Broadway North, Fargo, ND, 58122-0170, USA

**Keywords:** Powassan Virus encephalitis, Tick borne encephalitis, Intraparenchymal hemorrhage, Seizures

## Abstract

**Background:**

Powassan virus is a flavivirus related to eastern hemisphere’s tick-borne encephalitis viruses. It can cause a rare but potentially life-threatening disease including encephalitis.

**Case presentation:**

We report four cases of POWV infection in Minnesota and North Dakota with known exposure to tick bites in 2011. Our first case was an 18-year-old male who dramatically presented with seizure and headache with positive serum analysis for Powassan virus immunoglobulin M. The second case was a 60 year old gentleman with intraparenchymal hemorrhage and was diagnosed via cerebrospinal fluid analysis. Thirdly, a 61 year old male developed altered mental status and encephalitis. Our fourth patient was a 69 year old male who had headache and non-focal weakness who was diagnosed with serum analysis.

**Conclusion:**

Symptoms of Powassan virus infection ranged from headaches to seizures and severe neurological symptoms. This study serves to highlight the increased detection of Powassan virus infection in the central north United States. This report focuses on the increasing incidence that can lead to increasing efforts for raising awareness regarding this infection. There is a need for clinician vigilance and public attention due to its increasing detection, westward progression and varied clinical presentations.

## Background

Powassan virus (POWV) was first isolated in 1958 and since then, over 50 cases of Powassan virus (POWV) reported between 1958 and 2011 [1-4]. Between 2000 and 2005, there were confirmed cases in northern central United States including Michigan and Wisconsin showing spread of disease westward [4,5]. Over the last five years, there were only a total of 39 cases that have been reported to Centers for Disease Control and Prevention (CDC) [3].

We report a single institution experience of four cases of POWV infection in 2011 from a teaching hospital in North Dakota. This number is half of all cases from the whole country reported to the CDC in the prior year suggesting the need for public attention regarding the disease in high identification area due to the potential morbidity associated with the disease and lack of available treatment options [6].

## Case presentation

### Case 1

An 18-year-old male was evaluated in October of 2011 in northwestern Minnesota in consultation with neurology service for a significant headache for two days and one episode of seizure. He was at work, cutting trees, and was suspended from a harness, where he suddenly experienced clenching of his right upper extremity. He then had a witnessed generalized convulsive seizure while hanging in the air and required assistance to be lowered to the ground. He had an associated headache, with no meningismus. Computerized tomography scan (CT scan) of the brain was unremarkable. Treatment was deferred at that point, as it was a single isolated seizure event. Subsequently he noticed worsening of his headache. Magnetic Resonance Imaging (MRI) of the brain was performed and was unremarkable. A lumbar puncture (LP) was performed at outpatient neurology clinic showing cerebrospinal fluid (CSF) protein of 53 mg/dL (normal range (N) = 15-45 mg/dL), glucose – 66 mg/dL (N = 50-80 mg/dL), cell count -237 total nucleated cells (4 red blood cells (N = 0-5), differential – 8% lymphocytes and 92% segmenters), and negative herpes simplex virus (HSV)– polymerase chain reaction (PCR). He subsequently developed right-sided weakness, worsening headaches, and somnolence and was subsequently transferred to our facility to receive a higher level of care. Upon transfer to our facility, it was then reported that the patient had a tick bite. He was treated with antibiotics (ceftriaxone, vancomycin, ampicillin, acyclovir and doxycycline which were later de-escalated based on infectious work up). An electro encephalogram (EEG) performed during hospitalization was normal and he had no further witnessed seizures. Work-up for the more common tick-borne infections including anaplasmosis/ehrlichiosis (PCR and serology), lyme serology (non reactive), and babesiosis PCR were unremarkable. West nile virus (WNV) Immunoglobulin M (IgM) was negative. His initial serum IgM and CSF IgM was negative (10/2011) for the POWV but subsequent serum analysis (11/2011) was positive for POWV IgM by IgM antibody capture – enzyme linked immunosorbent assay (MAC-EIA) method. He was treated symptomatically. He gradually improved and was discharged. During his follow up visit he did not report any focal neurological deficits, seizures, or headaches.

### Case 2

**Figure 1:**
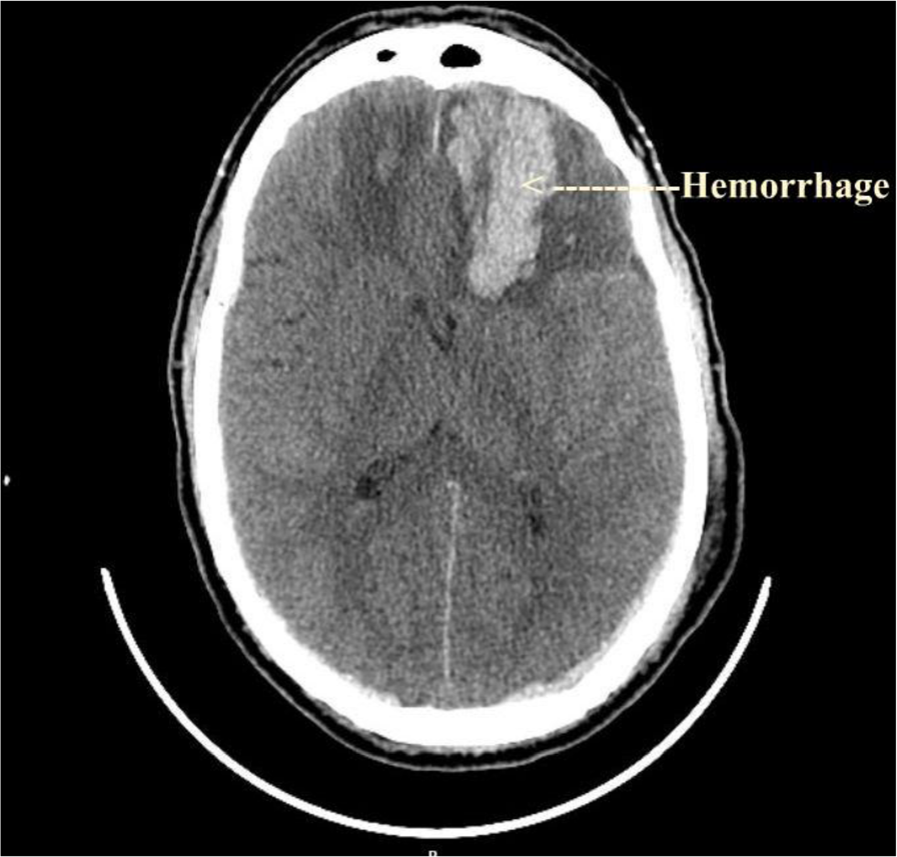
**CT scan brain image of CASE 2 at initial presentation in hospital showing acute intraparenchymal hemorrhage in the frontal lobe area.** This case suggests severity of presentation.

**Figure 2:**
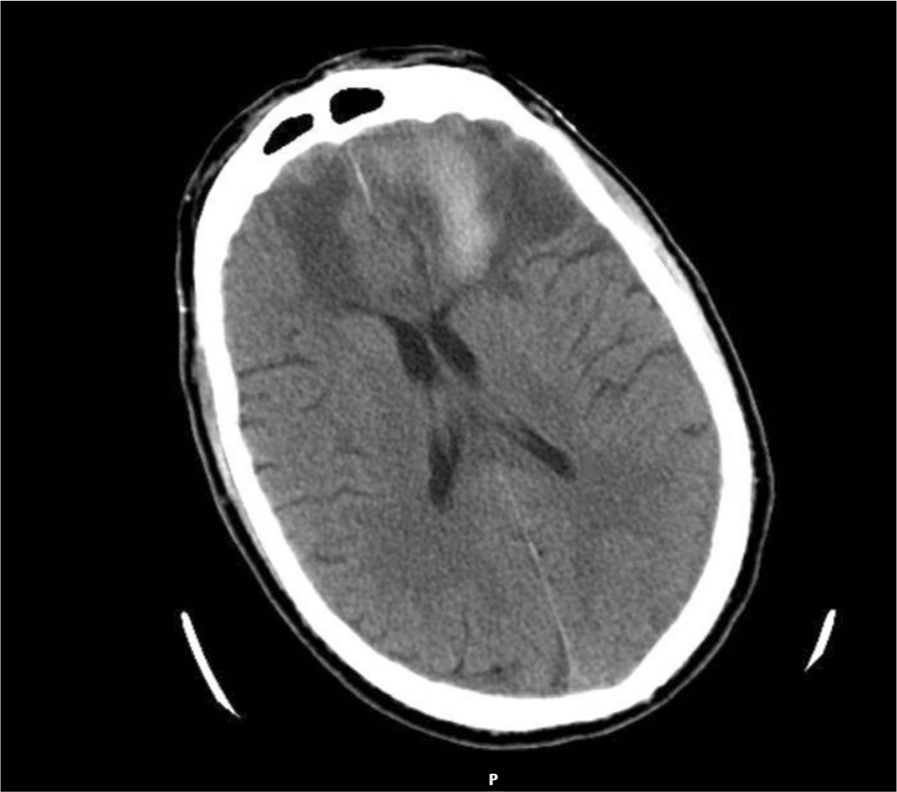
CT scan image of the brain of CASE 2 after 2 weeks of initial presentation suggesting partial resolution of the hemorrhage and no further progression or new hemorrhages coinciding with the clinical improvement.

**Figure 3:**
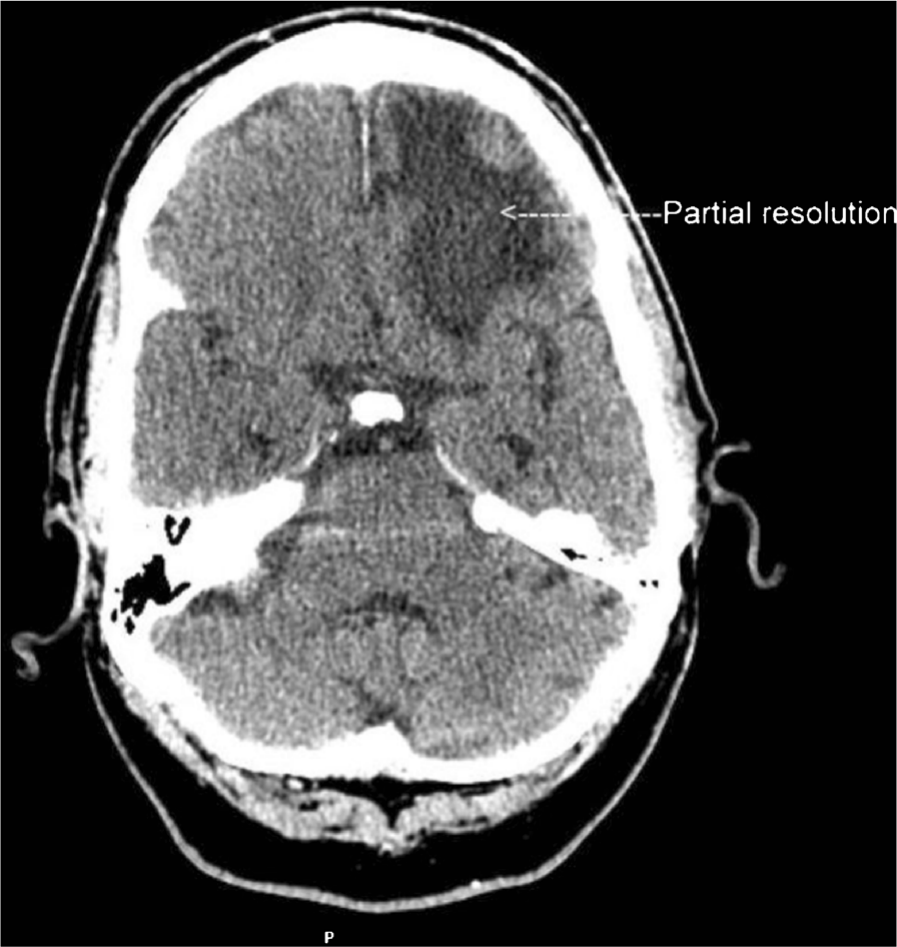
CT scan brain image of CASE 2 at 6 weeks of the presentation showing significant resolution of the hemorrhage with significant clinical improvement.

A 60-year old male who enjoys outdoor activity, with a history of colon cancer (treated with hemicolectomy and chemotherapy), diabetes mellitus and hypertension, presented to the emergency department with fevers, headaches and dizziness. His emergency department examination was unremarkable for focal neurological deficits, and he was discharged. Subsequently on his way to pharmacy, he lost consciousness and was found obtunded and unresponsive. He was intubated and transferred to the intensive care unit (ICU). He was afebrile upon admission. Initial evaluation included CT scan of the brain that suggested intraparenchymal hemorrhage with a subdural hematoma (Figure 1). His laboratories were unremarkable except for pancytopenia with a white blood cell (WBC) count of 2.3 × 10^3^/ul (N = 4.5 – 11.0 × 10^3^/ul), platelets of 81 × 10^3^/ul (N = 150-450 × 10^3^/ul) and hemoglobin of 10 g/dL (N = 13.5 -17.5 g/dL). Angiogram of the brain was unremarkable for vascular anomaly. CSF analysis was not performed due to multiple failed lumbar puncture attempts initially. He was stabilized and subsequently extubated after seven days. Upon transfer from the ICU, he did not have focal neurological deficits, but was found to have altered mental status, memory loss and anhedonia with depression. Subsequently his long hospital stay was complicated with central diabetes insipidus leading to hypernatremia that required vasopressin. He had a lumbar puncture on day 14 of hospitalization which revealed xanthochromia (113 RBCs/ul) with total of 12 WBC/ul which was predominantly lymphocytic (97%) and protein of 80 mg/dL (normal range (N) = 15-45 mg/dL), glucose – 166 mg/dL (N = 50-80 mg/dL). Infectious work up was negative for human immunodeficiency virus (HIV) in blood, syphilis serum rapid plasma regain (RPR), babesiosis serum PCR and peripheral smear, WNV IgM in serum, anaplasmosis/ehrlichiosis serum PCR and peripheral smear, varicella zoster virus CSF PCR, HSV CSF PCR, lyme disease serum and CSF serology, enterovirus CSF PCR, California encephalitis virus IgG and IgM [< 1:10 (N = < 1:10)], and Eastern equines encephalitis antibody IgG and IgM [< 1:10 (N = < 1:10)]. CSF fluid was positive for immunoglobulin G (IgG) titers of Saint Louis virus in high range (> = 1:40 with normal range <1:10) and also IgM titer positive for POWV detected via MAC-EIA (reported as detected IgM level in Minnesota Department of Health (MN DOH) laboratory). The positive POWV IgM test was confirmed by the National Institute of Infectious Diseases at CDC via serum dilution plaque reduction neutralization test (PRNT) suggesting titer of > 640 (Positive control results > 640). He was treated symptomatically. His subsequent imaging studies did not show any more progression of intraparenchymal hemorrhages and subsequent improvement (Figures 2, 3). Patient was subsequently transferred to a rehabilitation facility. His neurological function recovered with return of his baseline functional status on further follow up.

### Case 3

A 61-year old male, from northwestern Minnesota, with a history of diabetes mellitus, hypertension, progressive B cell lymphoma (multiple failed chemotherapy and stem cell transplantation), presented with progressive headaches, body aches, high-grade fever, and altered mental status. He enjoyed outdoor activities and reported frequent tick bites. Initial examination did not reveal focal neurological deficits. CT scan of the brain was unremarkable. Laboratory evaluation showed a WBC count of 2.5 × 10^3^/ul (N = 4.5 – 11.0 x10^3^/ul). Neoplastic involvement of central nervous system and infection were possible differential diagnosis for which an LP was performed. The initial CSF sample showed 3 WBC/ul, Protein 46 mg/dL (normal range (N) = 15-45 mg/dL), glucose – 57 mg/dL (N = 50-80 mg/dL). CSF was tested for HSV PCR Type 1/Type 2 (undetectable), WNV IgG (Results < 1.30, positive - >1.49) and WNV IgM (Result < 0.90, positive > 1.10), California encephalitis virus IgG and IgM (Result < 1:16, positive - >1:16), western equines encephalitis virus IgG and IgM (Result < 1:16, positive - >1:16), eastern equines encephalitis virus IgG and IgM (Result < 1:16, positive - >1:16), and St. Louis encephalitis virus IgG and IgM (Result < 1:16, positive - >1:16) The CSF sample was also checked for paraneoplastic autoantibody panel and neoplastic cells which were all negative. His fungal serology, cytomegalovirus PCR, babesia PCR, mycoplasma IgM screen, chlamydia serology, epstein-barr virus PCR and urine histoplasma antigen assay, urine blastomyces antigen, serum beta-D glucan (Value– 42 pg/ml – positive screen - >80 pg/ml) and sputum acid fast bacilli assay were all negative. Patient was initially treated with ceftriaxone, vancomycin and doxycycline. Lyme serology, anaplasmosis and ehrlichiosis serologies were negative. Due to clinical deterioration and hemodynamic instability patient was transferred to our facility where he was intubated and stabilized with mechanical ventilation. MRI of the brain was unremarkable. Antibiotics were changed to vancomycin, acyclovir, piperacillin/tazobactam and micafungin. Another LP was performed (5 days from initial LP). CSF was negative for coccidioidiomycosis antibody –IgG/IgM, histoplasma – yeast/mycelium/immunodiffusion, blastomycosis antibody by EIA, and fungal culture. The CSF was also sent to the Minnesota department of health (MN DOH) for POWV study. Palliative care was subsequently initiated due to lack of patient improvement initially and underlying lymphoma. Three weeks after his discharge to palliative care, his CSF (Second LP) was positive by PCR. He was eventually diagnosed with POWV encephalitis. Patient stayed in rehabilitation facility for over a month and eventually showed gradual improvement and was discharged home. His follow up serum POWV IgM analysis by MAC-EIA was negative (four months after initial presentation).

### Case 4

A 69-year old male with history of tick bites was evaluated at a local emergency room for progressive weak-ness, headaches and fevers. He was diagnosed with a urinary tract infection and was treated with ciprofloxacin. He continued to have fevers and progressive weakness with difficulty ambulating and subjective description of not able to control the movement of limbs. He presented to the emergency room again with weakness and a temperature of 100.4 F. His neurological examination was suggestive of non-focal generalized weakness but without any loss of muscle power. Laboratory workup suggested WBC of 12.8 × 10^3^/ul (N = 4.5 – 11.0 x10^3^/ul) and platelets of 90 × 10^3^/dL (N = 150-450 × 10^3^/ul)/dL. Initial infectious work up included Anaplasmosis/Ehrlichia serology for IgG antibody (Negative - <1:16), Ehrlichiosis/Anaplasmosis PCR (negative), West Nile IgM (negative), *Babesia microtti* IgM (< 1:16), Babesia microtti PCR (negative), and Lyme serology (non reactive). He was empirically treated with IV doxycycline. He continued to have fevers during his admission. POWV antibody panel was collected from serum. The patient gradually improved with return of his strength. He was subsequently discharged to a skilled nursing facility for further rehabilitation. After discharge from the hospital, IgM for POWV (MAC-EIA) was subsequently positive (3 weeks after presentation). He continued to improve neurologically. However, upon his last follow up at four months after his hospitalization he continued to have quadriceps muscle weakness and requires use of arm to get up from chair and with restriction of range of motion of lower limb at knee joint due to his weakness.

## Discussion

We report a total of four cases of Powassan virus encephalitis. Our first case presented dramatically with seizure and headaches with negative initial CSF analysis for POWV. Subsequently his serum analysis was positive for POWV IgM. He reported complete recovery from POWV encephalitis. The second case presented with intraparenchymal hemorrhage and was diagnosed via CSF analysis with positive IgM for POWV. He subsequently had full recovery. The same patient also had a positive IgG titer for the Saint Louis encephalitis virus. Our third patient developed altered mental status with negative initial CSF analysis for POWV but his serum analysis was positive for POWV IgM and on follow up CSF was positive for POWV. This immunocompromised patient with B-cell lymphoma had the most severe manifestation of the disease. Despite his severe neurological deficits, he did improve slowly, but has not returned to baseline neurological function. Our fourth patient had the least of the manifestations of POWV encephalitis having mainly headaches and non-focal weakness. He has almost fully recovered except for minor residual weakness of the quadriceps muscles. His disease was diagnosed with serum analysis that was positive for POWV IgM with continuous positivity after a month of presentation on repeat analysis. All four patients are reported to have significant outdoor exposure with reported tick bites. Overall, symptoms ranged from headaches to seizures and severe neurological symptoms.

POWV infection is acquired by tick bites usually Ixodes (Ix.) cookie, Ix. marxi, Ix. spinipalpus and Dermacentor andersoni ticks [7]. There are 38 mammal species identified as a domestic reservoir of POWV [4]. There is a high antibody prevalence of the disease on serological survey (1 to 4%) suggesting that there are higher rates of asymptomatic infection. After the tick bite the incubation period is reported to be around 8 to 34 days [8]. The spectrum of infection varies from nonspecific prodrome of upper respiratory symptoms leading to varied neurological involvement such as headache, dizziness, weakness, and hemiplegia [8]. Gholam et al and a few other researchers have reported a high case fatality rate of up to 5 to 10% with high rates of residual neurological dysfunction in the survivors including hemiplegia, memory impairment, and ophthalmoplegia [5,8-10].

Diagnosis of POWV encephalitis is established by following guidelines established by Center for disease control and prevention (CDC) and Council of State and Territorial Epidemiologists [11]. This specifies use of clinically compatible disease findings with laboratory confirmation of detecting IgM antibody against POWV in serum or CSF [11]. The serum test positivity is usually confirmed by Arbovirus Diagnostic Laboratory (ADL) at the CDC for additional testing like demonstration of PRNTs [4]. Reverse transcriptase polymerase chain reaction in CSF is also used as a diagnostic modality as reported in recent study from Minnesota [12]. In our study case 1 reported initial negative IgM analysis followed by a positive IgM analysis with MAC –EIA method. It does draw our attention to the fact that what may have caused such a difference. The authors are assuming that the variation in the laboratory work may be the result of either a longer lag phase in appearance of IgM antibody in serum or the second titer was high likely due to a positive convalescent titer. At this time we do not have any available research on time of detection of serum IgM after initial exposure.

Choi and colleagues recently reported a case of bilateral thalamic hemorrhagic encephalitis from POWV [13]. The patient reported in their case had resolution of the hemorrhage on follow up with clinical improvement. Case 2 reported here had bilateral frontal and temporal intraparenchymal hemorrhage on presentation and his clinical status did improve subsequently without radiological improvement after one month. Subsequent CT brain images after two months showed resolution of intraparenchymal hemorrhage (Figure 3). As mentioned in other literature there is no specific preferential area of hemorrhagic involvement within the brain in POWV encephalitis.

The disease is gradually making its way from the northeastern United States to the Midwest United States [2,4,5]. Though the cases reported here turned out to be non fatal, the disease has been reported to have a high case fatality rate [8]. Currently the serum IgM and CSF IgM tests are the commonly used diagnostic tests for the POWV detection [11]. The initial positive test from the state health department is confirmed by further testing in CDC.

One of the limitation of our study is the use of MAC –EIA method for the diagnosis of POWV. Most of our patients with compatible findings underwent sample collection, which was sent to MDOH for detection, and subsequent confirmation and long term follow up for resolution of titers. Due to the unavailability of the test commercially in most of the hospitals our diagnosis ability relied on testing done following the guidelines of CDC and we relied on the diagnosis based on what method the health department choose to diagnose the disease. In our case 3 the MN DOH used the reverse transcriptase PCR for the diagnosis of the patient.

There should be a higher clinical suspicion for the detection of POWV infection and that should lead to the ordering of appropriate diagnostic testing. The current obstacle to the detection of the disease is lack of clinical suspicion, and the lack of commercial diagnostic testing at regional hospitals.

There is no effective vaccine or treatment available for this clinical entity and the primary focus should be on prevention of acquiring the disease specifically through tick bites. Due to the lack of public awareness and lack of specialized testing with delay in diagnosis, the actual prevalence of POWV infection may be underreported. It may be beneficial to have public education to increase awareness for POWV infections in areas of high endemnicity. There should be systemic environmental efforts to identify the tick-infested areas with the POWV infection and all efforts should be performed to educate the population in that area to minimize the exposure.

## Conclusion

These four cases in a single year seen in our institution is the highest disease burden we have seen in the state of northern Minnesota and North Dakota. The actual rates of infection may be higher than reported due to the possibility of missed cases in the community. There is definitely a high level of exposure to ticks in the Midwest due to occupational and recreational exposures in the warmer months of the year with high risk associated of acquiring the disease. Our case series shows the importance of bringing this as a public health concern due to the significant tick exposures in the upper Midwest, which is known to be endemic for certain tick-borne infections. A larger ecological investigation in upper Midwest in form of Entomological survey may be helpful to identify the areas with high infestation for ticks with POWV. The ecological study can also address the co-infection rates in the ticks for Borellia burgdorferi, Anaplasmosis, and Ehrlichiosis, for which Ixodes is a common carrier. Information from these investigations can potentially lead to public awareness regarding POWV infections.

## Consent

Written informed consent was obtained from the patient for publication of this Case report and any accompanying images. A copy of the written consent is available for review by the Series Editor of this journal.

## Abbreviations

POWV: Powassan virus; CDC: Center for disease control; CT: Computerized tomography; MRI: Magnetic Resonance Imaging; LP: Lumbar puncture; CSF: Cerebrospinal fluid; HSV: Herpes simplex virus; PCR: Polymerase chain reaction; EEG: Electro encephalogram; WNV: West Nile virus; MAC: IgM antibody capture; EIA: Enzyme linked immunosorbent assay; MN DOH: Minnesota Department of health; PRNT: Plaque reduction neutralization test.

## Competing interests

Authors do not have any financial interest in any professional organization with competing interest.

## Authors' contributions

All authors have contributed equally in the chart review and manuscript write up. All authors read and approved the final manuscript.
